# Correlation between renal function and OCTA parameters of the retina and choroid in early-stage diabetic patients

**DOI:** 10.1186/s12967-025-07489-w

**Published:** 2025-11-25

**Authors:** Xindan Xing, Jin Wei, Yinchen Shen, Yuhang Ma, Liping Gu, Yufan Wang, Kun Liu, Li Su

**Affiliations:** 1 Department of Ophthalmology, Shanghai General Hospital, National Clinical Research Center for Eye Diseases, Shanghai Clinical Research Center for Eye Diseases, Shanghai Key Clinical Specialty, Shanghai Key Laboratory of Ocular Fundus Diseases, Shanghai Engineering Center for Visual Science and Photomedicine,Shanghai Engineering Center for Precise Diagnosis and Treatment of Eye Disease, Shanghai, China; 2 Department of Endocrinology and Metabolism, Shanghai General Hospital, Shanghai Jiao Tong University School of Medicine, No.100 Haining Road, Shanghai, 200080 China

**Keywords:** Swept-source optical coherence tomographic angiography, Retina, Choroid, Renal function, Early diabetic patients

## Abstract

**Background:**

To explore the associations between renal function and retinal/choroidal microvascular characteristics as well as structure through swept-source optical coherence tomographic angiography (SS-OCTA).

**Methods:**

This cross-sectional study recruited diabetic patients without diabetic retinopathy (DR) who visited the National Metabolic Management Center (MMC) in Shanghai General Hospital, Shanghai, China. The 6 mm×6 mm fundus images obtained by SS-OCTA were divided into three rings: a central ring (C, 1 mm), an inner ring (I, 3 mm), and an outer ring (O, 6 mm). The perfusion and vessel length density of the superficial and deep capillary plexus (SCP-PD, SCP-VLD, DCP-PD, DCP-VLD), foveal avascular zone (FAZ), macular thickness (MT), choroid thickness (CT), choroid volume (CV) and choroidal vascularity index (CVI) were evaluated. Renal function was reflected by the urinary microalbumin‒creatinine ratio (UACR), estimated glomerular filtration rate (eGFR), blood urea nitrogen (BUN) and the renin‒angiotensin‒aldosterone system (RAAS). Pearson correlation coefficients, multivariate analyses and interaction analyses were used to analyse the correlations between OCTA parameters and renal function.

**Results:**

eGFR was positively correlated with the SCP-PD (Average, C, I, O), SCP-VLD (Average, C, I), DCP-PD (C, I), DCP-VLD (C, I), CT (C, I, O, Overall) and CV (C, I, O, Overall). BUN was negatively correlated with the SCP-PD (C), SCP-VLD (C), DCP-PD (C, I), DCP-VLD (C), FAZ circulation. UACR was negatively correlated with the SCP-PD (C) and SCP-VLD (C). Angiotensin was negatively correlated with the SCP-PD (C), MT (C, I). According to the multivariate analysis, as chronic kidney impairment progressed, the SCP-PD (C) and SCP-VLD (C) decreased.

**Conclusion:**

There are significant correlations between renal function and retinal/choroidal structural and microvascular characteristics in diabetic patients without DR. These findings suggest that OCTA-derived parameters may serve as potential early biomarkers for diabetic microvascular complications.

**Supplementary Information:**

The online version contains supplementary material available at 10.1186/s12967-025-07489-w.

## Background

The incidence of diabetic retinopathy (DR) and diabetic nephropathy (DN) is increasing annually with the increasing prevalence of diabetes mellitus (DM), which poses a serious threat to public health [1, 2]. The microvascular disorders caused by hyperglycaemia, which lead to an increase in inflammatory factors and oxidative stress, are the common pathogenic mechanisms of DR and DN [3, 4]. Early detection of DR and DN is crucial, as early intervention can lead to better outcomes. However, the early detection and management of patients with DR and DN present significant challenges. One of the most important reasons is that extensive biochemical and imaging examinations, which are costly and partially invasive, are needed.

Optical coherence tomography angiography (OCTA) is a noninvasive three-dimensional (3D) imaging technique capable of vividly visualizing the capillaries in each layer of the retina [5, 6]. Compared with spectral domain OCTA (SD-OCTA), sweep source OCTA (SS-OCTA) features a faster imaging speed, a longer, deeper penetrating wavelength (1050 nm), and reduced sensitivity roll-off, which offers some advantages for reliable visualization and quantification of the choroid. A growing number of studies have confirmed that OCTA can serve as the earliest evidence for identifying microstructural and microvascular changes, such as an increased foveal avascular zone (FAZ) area, capillary nonperfusion areas, and reduced parafoveal vessel density in the deep capillary plexus (DCP), even before clinical signs appear [7, 8]. However, most of these studies were restricted to the detection of retinal microvascular changes because SD-OCTA equipment with lower penetrating ability and scanning speed was used. The detection of choroidal structural and vascular changes in diabetic patients without DR needs further elucidation.

Since microvascular disorders caused by hyperglycaemia are common pathogenic mechanisms of DR and DN, several previous studies have revealed a definite connection between DR and kidney disease [9–11]. Through fundus photography, Lim and colleagues discovered that narrower retinal arterioles, smaller fractal dimensions, focal arteriolar narrowing and opacification were associated with a greater probability of renal disease [9]. Moreover, several researchers have demonstrated that there is a significant positive correlation between the severity of kidney disease and the severity of DR by using fundus photography [10, 11]. However, compared with OCTA, fundus photography images cannot provide sensitive microvascular/microstructural metrics. Recent investigations have shown the potential of OCTA in monitoring systemic conditions, especially renal function [12–17]. Parameters derived from OCTA, such as attenuation of macular thickness, changes in perfusion patterns of the superficial and deep retinal vascular networks, and microvascular density [12, 17], have been found to strongly correlate with indicators of kidney disease, such as the estimated glomerular filtration rate (eGFR) and the urine albumin creatine ratio (UACR). Nevertheless, these studies focused mainly on patients who already had DR rather than those who had DM in the early stage without DR. There is currently no conclusion on whether there is a correlation between the changes in microvasculature and microstructure of the retina and renal function in early-stage diabetic patients without DR. Due to the penetrating limitation of SD-OCTA, these studies were restricted to detection of the retinal microvascular changes without the detection of choroid structural and vascular changes and concentrated on the correlation between renal function and the changes in retinal microvascular characteristics and microstructure. In these studies, the eGFR and UACR were the main indicators reflecting kidney function. Considering the promoting effect of renin‒angiotensin‒aldosterone system (RAAS) abnormalities on DR and DN, the correlation between the RAAS and retinal and choroidal microvascular and structural changes in early-stage DM patients needs further elucidation [18, 19].

Therefore, this study aimed to comprehensively analyse the correlation between renal function and microvascular characteristics, as well as the structure of the retina and choroid, in early diabetic patients without DR using SS-OCTA. This integrated approach may offer novel insights into early and noninvasive disease monitoring in diabetic patients.

## Methods

### Study design

This was a cross-sectional study involving type 2 diabetes patients who visited the National Metabolic Management Center (MMC) in Shanghai General Hospital, Shanghai, China, from May 12, 2021, to March 10, 2022. Diabetic patients without retinopathy were enrolled in the study provided that there was no media opacity interfering with photography or OCTA capture. DR patients were excluded according to the International Clinical Diabetic Retinopathy Severity Scales [19]. The additional exclusion criteria were as follows: (1) pregnancy; (2) any previous retinal or macular diseases, as well as glaucoma; and (3) an equivalent spherical lens greater than − 6.0 D or an optic axis longer than 26 mm. This exploratory cross-sectional study determined the sample size primarily based on the number of patients with diabetes but without DR who met the strict inclusion criteria during the study period (feasibility principle). Written informed consent was obtained from all participants prior to enrollment. The study was approved by the Ethics Committee of Shanghai General Hospital, Shanghai Jiao Tong University School of Medicine (no. 2017KY209) and was carried out in accordance with the principles of the Declaration of Helsinki [20].

All patients underwent a comprehensive ophthalmic examination, which included assessment of best-corrected visual acuity, slit-lamp biomicroscopy, fundus photography, and SS-OCTA. Basic personal information was collected from the electronic medical records system (HITELL.v3.0). Systolic and diastolic blood pressures were measured by experienced nurses using a blood pressure monitor (HBP-9020; OMRON, Osaka, Japan) following standard procedures. Body mass index (BMI) was calculated as weight (kg) divided by the square of height (m). Laboratory tests, including fasting blood glucose (FBG), glycosylated haemoglobin (HbA1c), serum creatinine (SCR), UACR, serum uric acid (SUA), blood urea nitrogen (BUN), renin, angiotensin, aldosterone, and other biochemical indicators, were performed according to standardized procedures in a certified laboratory in China. The eGFR was calculated via the simplified Modification of Diet in Renal Disease (MDRD) equation.

### OCTA measurements

All patients were imaged with the commercially available PLEX Elite 9000© OCTA (Zeiss Meditec, Dublin, CA) via a 6 × 6-mm scanning protocol while sampling at a 500 × 500 resolution at a rate of 100,000 A-scans per second. The A-scan depth was 3 mm, the axial resolution was 6.3 μm, and the transverse resolution was 20 μm, as listed in the product specifications. Images were acquired using the built-in fixation-tracking function, which detects and corrects for microsaccadic eye movement and blinking. Two consecutive 6 × 6 mm en-face images were acquired in series via the built-in registration function for each eye. En-face images of the superficial retinal capillary plexus (SCP), DCP, the choroidal vascularity were obtained through automatic layer segmentation by the device. The Angio slabs Retina (top ILM, bottom Bruch’s minus 41 microns), Superficial (top ILM, bottom IPL), and Deep (top IPL, bottom OPL) form the basis for subsequent quantification. The choroid is defined from Bruch’s membrane to the choroidal-scleral interface, whereby the boundaries are defined via Multilayer Segmentation. The magnification effect by axial length was adjusted using Littmann’s method and the Bennett formula [21].

To reduce the influence of potential confounders, a strict image quality criterion was implemented, where images labelled with less than 8/10 signal strength (as evaluated by proprietary Zeiss software) were not saved. Images of both eyes of each patient were collected, and two OCTA scans were performed for each eye. The scans with higher image quality were selected and included in our study. The majority of the images analysed had a signal strength score of 10/10. Images were manually inspected during acquisition for signs of improper focus and other artefacts, such as floaters. Two researchers evaluated image quality and segmentation accuracy, ensuring at least two high-quality images were acquired per eye. They visually inspected the generated topographic thickness map to confirm proper placement of the ETDRS grid and ensure no regions were incorrectly excluded from analysis. All segmentation boundaries (all B scans) were inspected, and manual correction was performed on any B-scans demonstrating segmentation errors before exporting data to the ARI Network. Used the overlay image to visually verify automated FAZ detection results before relying on numerical outputs.

All scans for each participant were conducted between 8:00–9:00 AM. This consistency in timing was specifically implemented to control for the known diurnal fluctuations in choroidal structures. Additionally, we implemented several other controls to minimize variation in choroidal measurements: participants were instructed to refrain from caffeine and nicotine consumption for at least two hours prior to measurements to prevent short-term alterations in choroidal thickness. A 10-minute resting period with distance viewing was incorporated prior to measurements. This served dual purposes: eliminating any accommodative choroidal thinning effects from near work and allowing blood pressure to stabilize.

All OCTA raw images were uploaded to the Zeiss Advanced Research and Innovation Network (www.arinetworkhub.com), and the website’s built-in algorithms were used to obtain analysis results. All uploaded images were divided into three rings using the ETDRS grid algorithm, with a central ring (C, 1 mm), inner ring (I, 3 mm), and outer ring (O, 6 mm). Macular thickness (MT) information was obtained via the ETDRS Retina Thickness v0.4 algorithm. The perfusion density of the SCP (SCP-PD) and DCP (DCP-PD); the vessel length density of the SCP (SCP-VLD) and DCP (DCP-VLD); and the raw length, circularity, and raw size values of the FAZ were obtained from the Macular Density v20210824-B algorithm. Information regarding the choroidal volume (CV), choroidal thickness (CT), and CVI was obtained through choroid quantification v20220224-B [22, 23]. CVI represents the ratio of the choroidal vessels (lumen volume) relative to the entire choroidal volume. The above algorithms are proprietary algorithm of the manufacturer. For more details, please refer to Supplementary Methods.

### Statistical analysis

Statistical analyses were conducted using SPSS V.22.0 for MAC and Python software. Descriptive statistics are presented according to variable characteristics: continuous variables are presented as the means ± standard deviations (means ± SDss), and categorical variables are presented as the counts and percentages [n (%), IQRs]. Dice similarity coefficients (DSC), intraclass correlation coefficients (ICC), the DSC served as our principal metric for evaluating inter-grader segmentation consistency.

The analytical approach followed a systematic procedure. First, Pearson correlation analysis was employed to evaluate bivariate correlations between OCTA parameters and laboratory test results, providing preliminary insights into potential associations between OCTA metrics and renal function markers. Age and sex adjustments were adjusted into the correlation analyses. Multivariate linear regression models were constructed to assess independent associations between OCTA parameters and renal function. eGFR was selected as the dependent variable, whereas participants’ demographic characteristics (age, sex, BMI), clinical biochemical indicators (e.g.HbA1c, lipids, inflammatory markers), refractive parameters (e.g.axial length, equivalent spherical diopter), usage of RAAS inhibitor and key OCTA metrics served as independent variables. Stepwise regression was applied to identify the optimal combination of variables while controlling for potential confounding factors. Model fitness was evaluated using adjusted R² values, and standardized regression coefficients (β) with 95% confidence intervals were calculated.

To validate the discriminative capacity of OCTA parameters for renal function status, participants were categorized into two groups according to clinical diagnostic criteria [24]: the non-CKD group (eGFR ≥ 90 mL/min/1.73 m² with no proteinuria, *N* = 108) and the CKD group (eGFR < 90 mL/min/1.73 m² or the presence of proteinuria, *N* = 56). On the basis of this classification, binary logistic regression analysis was performed to calculate associations between OCTA parameters and CKD risk, expressed as odds ratios (ORs) with 95% confidence intervals. The model similarly controlled for potential confounding factors, including age, sex, hypertension, diabetes, and other traditional risk factors.

To further identify important subgroups, we conducted a comprehensive exploratory interaction analysis between key clinical variables (age and glycemic control status) and major OCTA parameters (SCP-PD, DCP-PD, and CV).

To further evaluate the potential of OCTA parameters as biomarkers for the early detection of renal dysfunction, receiver operating characteristic (ROC) curve analysis was conducted, with optimal diagnostic thresholds determined using Youden’s index.

To control the risk of Type I errors arising from multiple comparisons, we applied the Benjamini-Hochberg (BH) procedure which preserves statistical power while still providing meaningful control over false positives for False Discovery Rate (FDR) correction to all correlation analyses. For the interaction analyses, we used the Bonferroni correction to strictly limit Type I error in the context of subgroup analyses to minimiz false-positive findings. Multicollinearity among predictor variables was evaluated using variance inflation factors (VIF) for both the linear regression model (Table 2) and logistic regression model (Supplementary Table S2). VIF values were calculated based on the correlation matrix of predictor variables. A VIF value < 5 was considered to indicate acceptable multicollinearity. For categorical variables entered as binary indicators, VIF was computed for the dummy-coded representation. Both SPSS and Python were utilized throughout to complete the statistical analyses and data processing.

## Results

In this research, 164 patients (164 eyes) with type 2 diabetes who met our inclusion criteria were recruited. Only one eye per patient was included in the study. The mean age was 57.39 ± 9.72 years, and 68% of the participants were male. The average body mass index (BMI) was 24.61 ± 4.54 kg/m², and the median duration of diabetes was 8.02 [0.6–12.3] years. The average systolic and diastolic blood pressures were 134.66 ± 18.54 mmHg and 79.62 ± 11.46 mmHg, respectively. Table 1 also presents a summary of the patients’ renal function outcomes and OCTA parameters.

**Table 1:** Patient demographics and clinical characteristics

Characteristics	164 T2DM Participants (164 Eyes)
**Demographics**	
Male, n (%)	112 (68.3%)
Mean age (y), mean ± SD	57.39 ± 9.72
Body mass index (kg/m^2^), mean ± SD	24.61 ± 4.54
Duration of diabetes (y)	8.02 [0.6,12.3]
Glycemic variability, mean ± SD	0.8 ± 0.4
Systolic blood pressure (mmHg), mean ± SD	134.66 ± 18.54
Diastolic blood pressure (mmHg), mean ± SD	79.62 ± 11.46
EF/BSA, mean ± SD	35.04 ± 9.11
Triglyceride (mmol/L), mean ± SD	1.65 ± 1.12
**Refractive Parameters**	
Axial Length (mm), mean ± SD	23.2 ± 1.2
Spherical Equivalent (D), mean ± SD	+ 0.5 ± 1.8
**OCTA parameters**	
SCP-perfusion density(%)	
Average-FOV, mean ± SD	40.09 ± 1.87
Central, mean ± SD	12.87 ± 4.61
Inner-ring, mean ± SD	37.30 ± 0.29
Outer-ring, mean ± SD	41.49 ± 1.92
SCP-Vessel length density (mm/mm^2^)	
Average-FOV, mean ± SD	22.0778 ± 1.2071
Central, mean ± SD	7.4758 ± 2.6342
Inner-ring, mean ± SD	21.4707 ± 1.8073
Outer-ring, mean ± SD	22.6787 ± 1.3641
DCP-perfusion density(%)	
Average-FOV, mean ± SD	36.09 ± 3.73
Central, mean ± SD	16.16 ± 4.95
Inner-ring, mean ± SD	36.88 ± 3.52
Outer-ring, mean ± SD	36.43 ± 3.97
DCP-Vessel length density (mm/mm^2^)	
Average-FOV, mean ± SD	20.6182 ± 2.4523
Central, mean ± SD	9.0169 ± 2.7693
Inner-ring, mean ± SD	21.2774 ± 2.1591
Outer-ring, mean ± SD	21.5501 ± 2.6058
FAZ Parameter	
FAZ raw length (mm), mean ± SD	2.2795 ± 0.5461
FAZ circularity, mean ± SD	0.6580 ± 0.0911
FAZ raw size (mm^2^), mean ± SD	0.2716 ± 0.1096
Macular Thickness (mm)	
Central, mean ± SD	254.3260 ± 19.9834
Inner-ring, mean ± SD	322.5472 ± 30.5986
Outer-ring, mean ± SD	281.3221 ± 26.1122
Overall, mean ± SD	285.2979 ± 13.2389
Choroid Volume (mm^3^)	
Central, mean ± SD	0.2133 ± 0.0866
Inner-ring, mean ± SD	1.6852 ± 0.6396
Outer-ring, mean ± SD	5.1018 ± 1.7264
Overall, mean ± SD	8.8657 ± 2.2134
Choroid Thickness (mm)	
Central, mean ± SD	274.2518 ± 102.25
Inner-ring, mean ± SD	270.5595 ± 101.83
Outer-ring, mean ± SD	248.85 ± 84.35
Overall, mean ± SD	248.19 ± 84.09
CVI	0.5848 ± 0.0207
**Laboratory Tests Results**	
HbA1c (%), mean ± SD	8.03 ± 2.01
**Renal Function**	
Serum creatine (µmol/L), mean ± SD	64.08 ± 19.94
Serum uric acid (µmol/L), mean ± SD	320.20 ± 88.31
Blood urea nitrogen (mmol/L), mean ± SD	5.38 ± 1.21
eGFR (ml/min/1.73/m^2^), mean ± SD	99.28 ± 17.82
UACR(mg/g)median [IQR]	10.79[5.53–20.86]
**RAAS**	
Renin (ng/mL), mean ± SD	23.39 ± 18.76
Angiotensin (pg/mL), mean ± SD	118.67 ± 17.25
**Inflammatory Markers**	
IL-6 (pg/mL), mean ± SD	3.67 ± 1.82
CRP (mg/L), mean ± SD	3.47 ± 2.21
ESR (mm/h), mean ± SD	14.05 ± 5.07
PCT (pg/mL), mean ± SD	0.08 ± 0.052
**Medication Use**	
RAAS inhibitor use, n (%)	n (%) 45 (27%)
Lipid-lowering agents use	n (%) 85 (52%)

OCTA: optical coherence tomography angiography; SCP: superficial capillary plexus; FOV: Field of view; DCP: deep capillary plexus; FAZ: the foveal avascular zone; HbA1c: glycated hemoglobin; eGFR: estimated glomerular filtration rate; RAAS: Renin-Angiotensin-Aldosterone System; UACR: Urinary microalbumin to creatinine ratio; EF/BSA: the ejection fraction to body surface area ratio; CRP: C-reactive protein; ESR: Erythrocyte sedimentation rate; IL-6: interleukin-6; PCT: procalcitonin

**Fig. 1:**
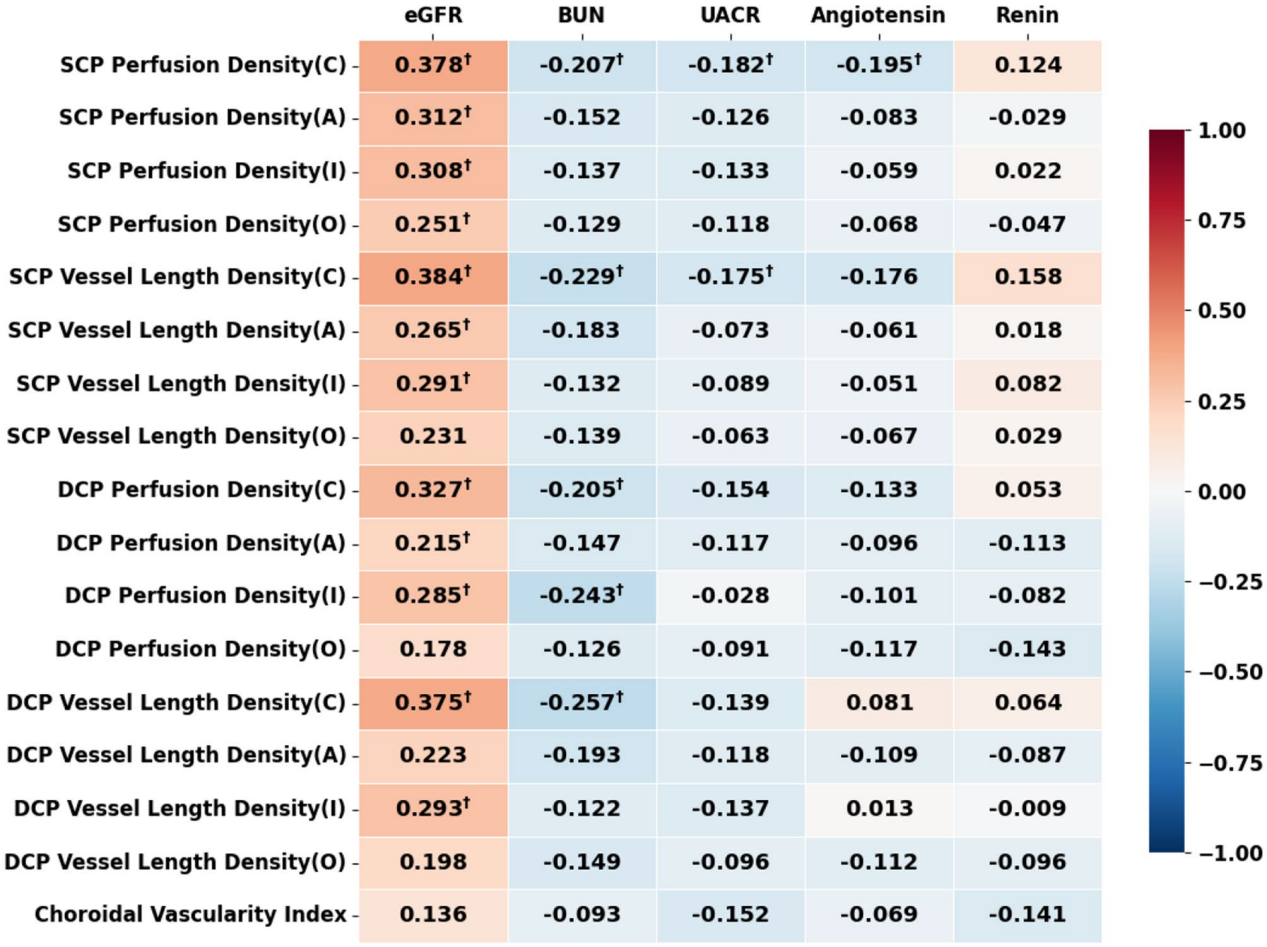
Heatmap of Pearson correlation coefficients between renal function and microcirculation of retina and choroid. Figure 1 presents the correlations between various retinal microcirculation parameters (including perfusion density and vessel length density of the SCP and DCP) and multiple renal function indicators (such as eGFR, BUN, UACR, angiotensin, and renin). Red indicates positive correlation, blue indicates negative correlation, and the color intensity represents the strength of correlation. To control for multiple comparisons, Benjamini-Hochberg procedure was applied to adjust p values. † indicates correlations that remained significant after FDR correction (*q* < 0.05). Abbreviations: SCP: superficial capillary plexus; DCP: deep capillary plexus; (C): Central-ring; (A): Average-region; (I): Inner-ring; (O): Outer-ring; eGFR: estimated glomerular filtration rate; UACR: Urinary microalbumin to creatinine ratio; CVI: Choroidal Vascularity Index; BUN: Blood Urea Nitrogen; RAAS: Renin-Angiotensin-Aldosterone System

Figure 1 presents the Pearson correlation coefficients between renal function and the microvascular characteristics of the retina and choroid. To balance the risk of Type I errors (incorrectly rejecting true null hypotheses) against Type II errors (incorrectly accepting null hypotheses), we selected the BH correction. This more conservative approach helps mitigate the risk of false positives while maintaining reasonable sensitivity to detect relevant associations.

After BH correction, eGFR had the most significant positive correlations with the SCP-PD (Average, C, I, O), SCP-VLD (Average, C, I), DCP-PD (Average, C, I), DCP-VLD (C, I). The correlation coefficients ranged from 0.215 to 0.384, with a particularly strong presence in the central ring (all pcc > 0, *q* < 0.05). Interestingly, highly prominent negative correlations were identified between BUN and the SCP-PD (C), SCP-VLD (C), DCP-PD (C, I), DCP-VLD (C), FAZ circulation (all pcc < 0, *q* < 0.05). With respect to UACR, we found strong negative associations between UACR and the SCP-PD (C) and SCP-VLD (C) (all pcc < 0, *q* < 0.05). Additionally, there were negative correlations between angiotensin and the SCP-PD (C) (pcc < 0, *q* < 0.05). There was no significant correlation between the CVI and renal function. All correlations that maintained significance after correction are marked with “ † ” in Fig. 1 and Supplementary Figure S1.

**Fig. 2:**
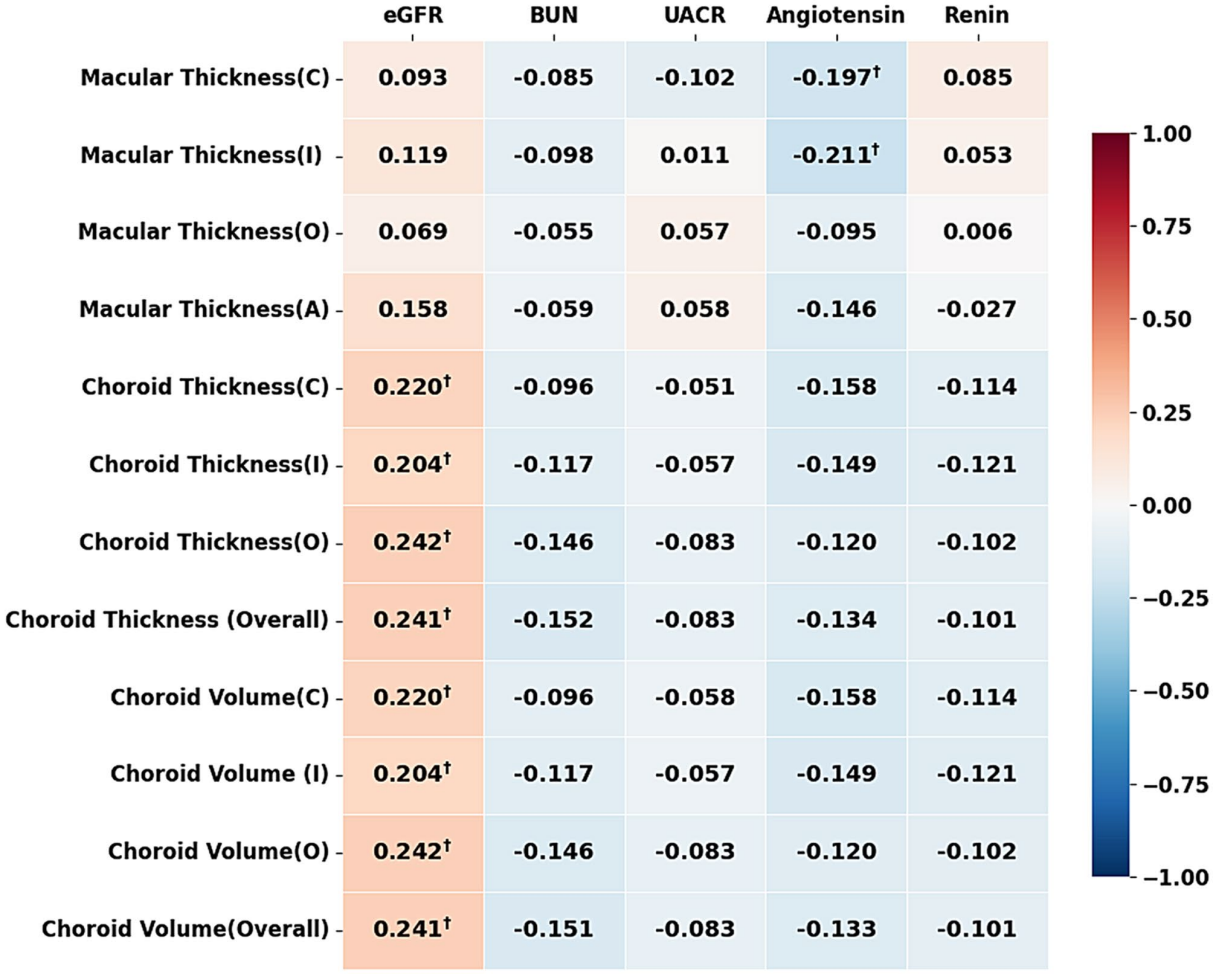
Heatmap of Pearson correlation coefficients between renal function and structure of retina and choroid. Figure 2 presents the correlations between various retinal microcirculation parameters (including macular thickenss, choroid thickness and volume) and multiple renal function indicators (such as eGFR, BUN, UACR, angiotensin, and renin). Red indicates positive correlation, blue indicates negative correlation, and the color intensity represents the strength of correlation. To control for multiple comparisons, Benjamini-Hochberg procedure was applied to adjust p values. † indicates correlations that remained significant after FDR correction (*q* < 0.05). The structural parameters are organized by measurement type (macular thickness, choroid thickness, and choroid volume) and region: Central (C), Inner-ring (I), Outer-ring (O), and Average/Overall (A/Overall). Abbreviations: eGFR: estimated glomerular filtration rate; BUN: blood urea nitrogen; UACR: urinary microalbumin to creatinine ratio

Figure 2 shows the Pearson correlation coefficients between renal function and the structure of the retina and choroid. After BH correction, the eGFR had the most significant positive correlations with CT and CV in all grids, with values ranging from 0.204 to 0.242 (all pcc > 0, *q* < 0.05). In contrast, there was no significant correlation between the eGFR and MT (all *p* > 0.05). Furthermorer, Angiotensin was negatively correlated with MT (C, I) (all pcc < 0, *q* < 0.05). All correlations that maintained significance after correction are marked with “ † ” in Fig. 2.

Given that the eGFR is a well-recognized indicator of renal function and that there was a significant correlation between OCTA parameters and the eGFR (Fig. 1), when selecting independent variables, we applied a two-fold approach: first, we prioritized OCTA parameters that demonstrated stronger correlation coefficients with eGFR in univariate analysis; second, we carefully evaluated the interrelationships among these parameters to minimize multicollinearity in our predictive model. Additionally, we included potential confounding factors that might influence renal function, such as age, BMI, the ejection fraction to body surface area ratio (EF/BSA), blood pressure parameters, glycaemic control indicators, usage of RAAS inhibitors and lipid metabolism markers, inflammatory markers, refractive parameters. Prior to constructing the final model, we conducted multicollinearity analysis among all the variables, calculating VIF values to identify and exclude highly correlated variables, ensuring that our regression model contained independent predictors without problematic multicollinearity (all VIF values < 5, Table 2). We found that a higher eGFR was still significantly associated with higher SCP-PD (C), DCP-PD (C) and was significantly correlated with younger age, lower BMI, lower TG, lower HbA1c, lower interleukin-6 (IL-6), shorter diabetes duration (all *p* < 0.05) (Table 2).

**Table 2:** Associations (multivariable regression) between eGFR and OCTA parameters

	Standardized Coefficients	95% confidence interval for B	*p*-Value	VIF
	Beta			
Age	-0.217	-1.148, -0.732	<0.001**	1.483
BMI	-0.147	-0.917, -0.318	0.008*	1.120
TG	-0.163	-0.831, -0.678	0.007*	1.104
SBP	-0.143	-0.158, -0.010	0.023*	1.045
DBP	-0.026	-0.047, 0.032	0.334	1.023
HbA1c	-0.163	-1.835, -0.428	< 0.001**	1.082
SCP-PD (C)	0.224	1.562, 3.813	< 0.001**	1.292
DCP-PD (C)	0.185	0.918, 3.726	0.042*	1.288
CV (Overall)	0.098	-1.312, 5.647	0.348	3.345
Diabetes duration	-0.167	-0.864, -0.239	< 0.001**	1.308
IL-6	-0.134	-0.847,-0.123	0.041*	2.451
EF/BSA	-0.067	-0.534, 0.156	0.389	1.189
Axial Length	0.098	-0.187, 1.456	0.178	1.345
Spherical Equivalent	-0.056	-0.634, 0.289	0.423	1.267
Glycemic variability	-0.112	-0.634, 0.089	0.156	1.423
RAAS inhibitor use	-0.112	-7.217, 1.034	0.138	1.285
Lipid-lowering agents	-0.093	-6.127, 1.433	0.217	1.238

R2: 0.536; Adjusted R2: 0.517

All VIF values < 5 indicate acceptable levels of multicollinearity, with no evidence of redundant predictors in the model

BMI: Body mass index; TG: Triglyceride; SBP: Systolic blood pressure; DBP: Diastolic blood pressure; HbA1c: Glycated hemoglobin A1c; SCP: Superficial capillary plexus; PD: Perfusion density; DCP: Deep capillary plexus; C:Central; EF/BSA: the ejection fraction to body surface area ratio; IL-6: interleukin-6

**Fig. 3:**
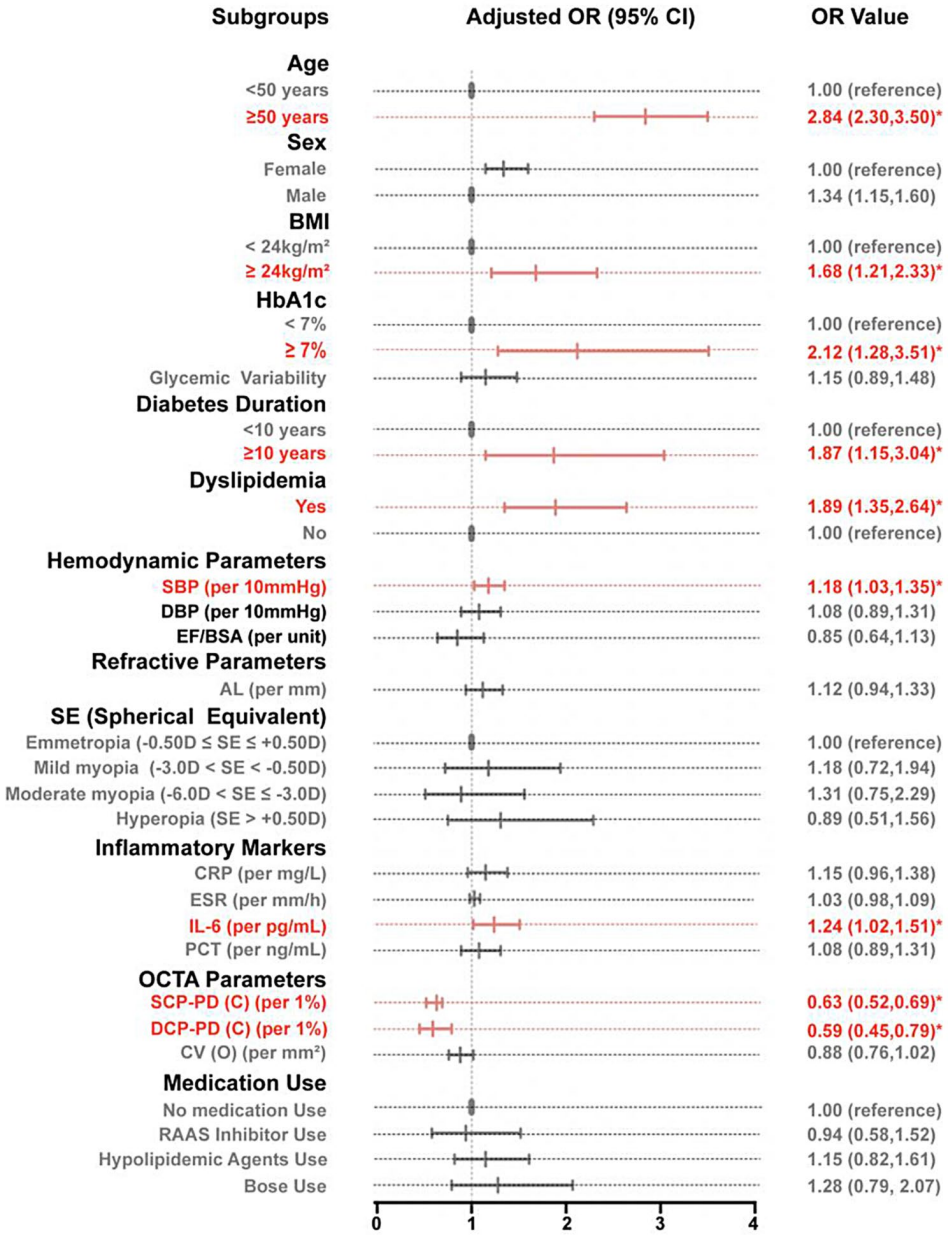
Logistic regression results between renal dysfunction and OCTA parameters. Figure 3 shows logistic regression analysis results of risk factors for renal dysfunction. The forest plot displays the strength of associations between various factors and renal dysfunction. Points and horizontal lines represent odds ratios (ORs) and 95% confidence intervals, respectively. Factors to the left of the vertical dashed line (OR = 1) are protective factors (OR < 1), while those to the right are risk factors (OR > 1). Red indicates statistically significant associations (*p* ≤ 0.05), and gray indicates non-significant associations. Abbreviations: OR: odds ratio; CI: confidence interval; BMI: body mass index; SBP: systolic blood pressure; DBP: diastolic blood pressure; HbA1c: glycated hemoglobin; RAAS: renin-angiotensin-aldosterone system; SCP: superficial capillary plexus; DCP: deep capillary plexus; PD: perfusion density; C: central; EF/BSA: the ejection fraction to body surface area ratio; CRP: C-reactive protein; ESR: Erythrocyte sedimentation rate; IL-6: interleukin-6; PCT: procalcitonin

Figure 3 presents the results of multivariate logistic regression analysis of risk factors associated with renal function impairment. After adjusting for potential confounding factors, we evaluated the independent associations between various clinical and imaging parameters and renal dysfunction. Multicollinearity assessment confirmed no problematic collinearity among predictors, with all VIF values < 5 (Supplementary Table S2). Among microvascular parameters, SCP-PD (C) emerged as a significant protective factor against renal function impairment (OR = 0.63, 95% CI: 0.52–0.69, *p* < 0.05), indicating that for each 1% increase in SCP-PD (C), the odds of renal dysfunction decreased by approximately 37%. Similarly, DCP-PD (C) demonstrated a protective association (OR = 0.59, 95% CI: 0.45–0.79, *p* < 0.05). In contrast, CV (Overall) showed no significant relationship with renal function status (OR = 0.88, 95% CI: 0.76–1.02; *p* > 0.05).

In addition to microvascular characteristics, age emerged as one of the strongest independent risk factors for renal function impairment, with an adjusted OR of 2.84 (95% CI: 2.30–3.50, *p* < 0.05), indicating that advancing age significantly increases the risk of renal dysfunction. Similarly, diabetes duration showed a substantial increase in risk (OR = 1.87, 95% CI: 1.15–3.04, *p* < 0.05), suggesting a cumulative detrimental effect of prolonged diabetes status on kidney function.

Analysis of metabolic parameters revealed that HbA1c levels were significantly associated with an increased risk of renal function impairment (OR = 2.12, 95% CI: 1.28–3.51, *p* < 0.05), emphasizing the importance of glycaemic control in renal protection. At the same time, glycemic variability demonstrated a positive trend with renal dysfunction risk but failed to achieve statistical significance (OR = 1.15, 95% CI: 0.89–1.48, *p* > 0.05), suggesting that long-term glycemic control may be more important than short-term glucose fluctuations in early-stage diabetic patients. An increased BMI was also significantly associated with a greater risk of renal impairment (OR = 1.68, 95% CI: 1.21–2.33, *p* < 0.05).

Among blood pressure indicators, SBP, although with a relatively modest association strength, reached statistical significance (OR = 1.18, 95% CI: 1.03–1.35; *p* < 0.05), suggesting that even slight elevations in blood pressure may increase the risk of kidney damage. However, DBP was not significantly associated with renal function impairment (OR = 1.08, 95% CI: 0.89–1.31; *p* > 0.05). Besides DBP, we incorporated EF/BSA as an indirect indicators reflecting the systemic blood flow status, the results showed that EF/BSA not significantly associated with renal function impairment (OR = 0.85, 95% CI: 0.64–1.13; *p* > 0.05).

Analysis of inflammatory markers revealed that IL-6 demonstrated statistical significance as an independent risk factor for renal dysfunction (IL-6 per pg/mL, OR = 1.24, 95% CI: 1.02–1.51, *p* < 0.05), which aligns with previous studies demonstrating that elevated IL-6 levels are associated with progressive diabetic nephropathy and correlate with both structural and functional renal changes in early-stage diabetes [25, 26], other inflammatory markers: C-reactive protein (CRP per mg/L, OR = 1.15, 95% CI: 0.96–1.38), erythrocyte sedimentation rate (ESR per mm/h, OR = 1.03, 95% CI: 0.98–1.09), and procalcitonin (PCT per ng/mL, OR = 1.08, 95% CI: 0.89–1.31) did not reach the significance criterion.

In addition to the above indicators, we also included gender, axial length, spherical equivalent, and medication use in the model. The results showed that these indicators did not reach statistical significance (Fig. 3).

Considering that testing for interactions between key variables could reveal important subgroups where OCTA metrics demonstrate enhanced predictive value, which represents a crucial analytical consideration for optimizing clinical utility, we conducted a comprehensive exploratory interaction analysis examining the relationships between key clinical variables (age and glycemic control status) and major OCTA parameters (SCP-PD (C), DCP-PD (C), and CV(Overall)). We tested six interaction terms and observed distinct patterns of effect modification across different OCTA parameters. Given that we tested six interaction terms simultaneously, we applied Bonferroni correction. After correction, none of the interactions achieved statistical significance (adjusted p-values ranging from 0.192 to 1.000). The results are summarized in Table 3.

**Table 3:** Exploratory interaction analysis between key clinical variables and OCTA parameters

Interaction Term	β Coefficient	95% CI	Original *p*-value	Adjusted *p*-value*	Statistical Significance
Age×SCP-PD(C)	+ 0.187	0.043–0.331	0.032	0.192	NS after correction
Age×DCP-PD(C)	+ 0.134	-0.018–0.286	0.089	0.534	NS after correction
Age×CV(Overall)	+ 0.098	-0.038–0.234	0.156	0.936	NS after correction
HbA1c×SCP-PD(C)	-0.156	-0.298 - (-0.014)	0.041	0.246	NS after correction
HbA1c×DCP-PD(C)	-0.119	-0.251–0.013	0.067	0.402	NS after correction
HbA1c×CV(Overall)	-0.087	-0.223–0.049	0.203	1.000	NS after correction

HbA1c: Glycated hemoglobin A1c; SCP: Superficial capillary plexus; PD: Perfusion density; DCP: Deep capillary plexus; CV: Choroid Volume; C: Central

**Fig. 4:**
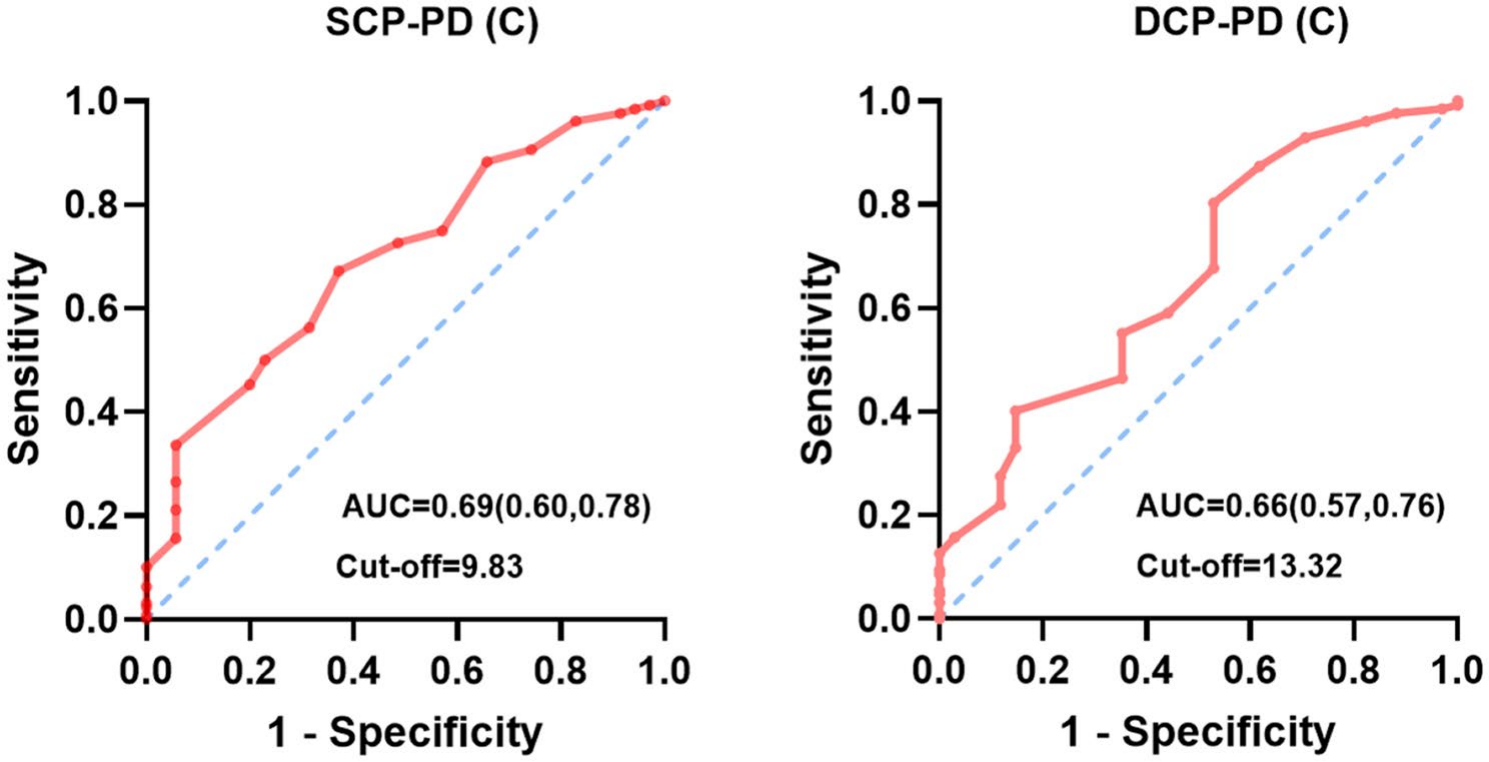
Receiver operating characteristic curves of renal dysfunction using the perfusion density of the central-ring retina in early-stage diabetic patients. Figure 4 displays the receiver operating characteristic curves for two retinal microcirculation parameters in predicting diabetic kidney dysfunction. The left panel shows the performance of the perfusion density of the superficial capillary plexus in central ring of retina (SCP-PD (C)) with an area under the curve (AUC) of 0.69 (95% CI: 0.60–0.78) and an optimal cut-off value of 9.83. The right panel presents the perfusion density of the deep capillary plexus in central ring of retina (DCP-PD (C)) with an AUC of 0.66 (95% CI: 0.57–0.76) and an optimal cut-off value of 13.32. The red lines represent the ROC curves, while the blue dashed diagonal lines indicate the reference line of no discrimination (AUC = 0.5)

Notably, our multivariate analysis revealed that, among the various OCTA parameters examined, the SCP-PD (C) and DCP-PD (C) measurements demonstrated the most promising predictive value (Fig. 3). In the ROC curve analysis (Fig. 4), for the SCP-PD (C), the area under the curve (AUC) was 0.69. At this point, the optimal cut-off value determined for the SCP-PD (C) was 9.83, indicating that diabetic patients without retinopathy who have an SCP-PD (C) less than 9.83 should be considered at greater risk for kidney function impairment. Similarly, the DCP-PD (C) had an AUC of 0.66, with an optimal cut-off value of 13.32. These findings collectively support the potential utility of central retinal perfusion parameters as noninvasive biomarkers for early renal dysfunction.

## Discussion

This study investigated the correlation between renal function and the structure and microvascular characteristics of the retina and choroid in diabetic patients without retinopathy through SS-OCTA. There were several main findings in the early stage of diabetes: (1) eGFR is positively correlated with retinal microvascular characteristics and the choroidal structure in almost all grids and layers. (2) BUN is negatively correlated with retinal microvascular characteristics in central ring of the retina. (3) UACR is negatively correlated with SCP-PD and SCP-VLD in central ring of the retina. (4) Angiotensin has a negative correlation with the SCP-PD (C), MT (C, I). (5) Our multivariate analysis identified several independent risk factors for renal function impairment, including decreased SCP-PD (C), decreased DCP-PD (C), advanced age, longer diabetes duration and metabolic abnormalities (elevated HbA1c, TG, IL-6, and BMI). These findings suggest that the development of renal dysfunction involves multiple pathological mechanisms, including age-related changes, metabolic dysregulation, inflammation and microvascular pathology and that SS-OCTA examination, especially focusing on perfusion density, may has the potential to identify diabetic patients who may require more comprehensive kidney function testing, thereby enabling early intervention before clinical symptoms of diabetic nephropathy become apparent.

The eGFR is an accurate indicator for evaluating renal function and is used to assess the excretion efficiency of toxins in the body by the kidneys. In the literature, the relationship between the eGFR and retinal microcirculation remains a matter of contention. Xu et al. reported that the eGFR was not significantly related to the macular vessel diameter, FAZ, or macular vessel area density [27]. Conversely, Yao et al. reported a significant positive correlation between the eGFR and superficial capillary plexus vessel density [28]. Similarly, Wei et al. reported a positive correlation between the eGFR and vessel density [14]. In contrast, Qi and colleagues reported that the eGFR was inversely correlated with vessel density in the nasal region of the deep capillary plexus layer and the supratemporal region of the mid-large choroidal vessel layer [29]. In the current investigation, we demonstrated highly significant positive correlations between the eGFR and the SCP-PD, SCP-VLD, DCP-PD, and DCP-VLD across all grids and layers. However, no significant associations were detected between the eGFR and retinal thickness or the FAZ area. In contrast, Yao H et al. reported a significant negative correlation between the eGFR and FAZ area, whereas Qi Z et al. reported a positive relationship between the eGFR and inner retinal thickness in the total, superior, central macular area, and inferior regions, as well as the choroidal thickness in the total, inferior, nasal, and inferonasal regions [28, 29]. We believe that the main reason for these inconsistent results is the inconsistent inclusion of populations. That is, whether the diabetic patients included in these studies had DR was not uniform. The research results of Yao et al. and Wei et al. are consistent with our findings. The populations included in these two studies are also consistent with ours, which are either all patients with diabetes but without DR (Yao et al.) or most patients with diabetes but without DR (Wei et al.). However, in the studies by Xu et al., non-DM patients, pre-DM patients, and DM patients (with or without DR) were included, and the eGFRs of these patients were all within the normal range. This could easily explain their different results. We have noticed that the population included in the study by Yao et al. is consistent with ours, that is, early-stage diabetic patients without DR. Their study found that there is a negative correlation between eGFR and the area of the foveal avascular zone (FAZ). However, in our study, no significant correlation between eGFR and the FAZ area was found. We speculate that the possible reasons are as follows: (1) The sample size in our study is approximately twice that in the study by Yao et al.; (2) Different OCTA devices were used. Yao et al. used the SD-OCTA AngioVue 2.0, while our study used the SS-OCTA PLEX Elite 9000©. Compared with SD-OCTA, SS-OCTA has a faster imaging speed, a longer penetration wavelength, a deeper penetration depth, and a smaller sensitivity roll-off. The above two points may be the reasons for the inconsistent results.

We also surprisingly found positive correlations between the eGFR and CT (C, I, N, overall) and between the eGFR and the CV (C, I, N, overall), which is consistent with the findings of previous studies [30, 31]. The choroid is a network composed of blood vessels, collagen fibres, fibroblasts and melanocytes, and changes in the thickness of the choroid are considered indicators of the impact of systemic diseases on the blood vessels of the eyes. The eGFR affects the distribution of blood throughout the body, and the blood supply to the choroid depends on the systemic blood circulation system. Therefore, it is reasonable that there is a correlation between the eGFR and the thickness and volume of the choroid.

The UACR is an indicator of glomerular injury and is used to assess whether there is damage to the renal filtration system. Pathological changes in podocytes or the basement membrane can lead to an increase in proteinuria and a rise in the UACR. In addition to the eGFR, we demonstrated a significant negative correlation between the UACR and retinal microcirculation in the central ring, which corroborates previous findings [11, 32, 33]. Zhang and colleagues reported a positive correlation between the UACR and retinal thickness [11]. However, in our study, no such association was observed between the UACR and the structure of the retina or choroid. This discrepancy might be because we enrolled early-stage diabetic patients without retinopathy, whereas Zhang H et al. included patients with DR whose retinal thickness had already been altered [11].

The BUN is the end product of protein metabolism and can reflect the metabolic status of the body’s proteins to a certain extent. The accumulation of blood urea nitrogen in the body indirectly reflects an increase in toxins (nitrogenous wastes) in the body. We established a significant negative correlation between BUN and retinal microvascular characteristics. Cai et al. and Wang et al. reported a positive correlation between BUN and the progression of DR [34, 35]. As DR progresses, retinal microvascular perfusion decreases. Thus, our results are consistent with those of prior studies. However, given that BUN is vulnerable to factors such as diet, it is not suitable for use as a sole indicator of renal function. Instead, it can serve as an adjunct to the eGFR and UACR to comprehensively reflect renal function.

The RAAS, one of the most intensively studied hormonal systems in the human body and has a long history of research, is widely acknowledged for its vital role in regulating systemic vascular tone and maintaining electrolyte balance [36]. Hyperglycaemia triggers the overexpression of RAAS components through multiple mechanisms, such as advanced glycation end products (AGEs), G-protein-coupled receptor 91 (GPR91), and the (pro)renin receptor ((P)RR) [37]. The RAAS can contribute to the progression of DR by increasing the expression of vascular endothelial growth factor (VEGF), increasing oxidative stress, and promoting inflammation [37]. In our study, we found that the level of angiotensin in the bloodstream was negatively correlated with the retinal microvascular characteristics, retinal structure, and choroidal structure. However, renin did not significantly affect either the retinal microvascular characteristics or the retinal structure. Our findings suggest an “effector-centric” activation pattern of the RAAS in early diabetes. Rather than the classical renin–angiotensin–aldosterone sequence, non-canonical pathways may directly generate Ang II, making it the most closely correlated effector with OCTA-detected microvascular changes. The observed dissociation between renin and Ang II may reflect strong negative feedback, where elevated Ang II suppresses renin release, and the fact that renin primarily responds to systemic hemodynamic demands rather than local retinal injury. In contrast, aldosterone showed no significant correlation, likely due to its multifactorial regulation (e.g., potassium, sodium, ACTH) and delayed genomic effects, which may not align with the relatively immediate microvascular states captured by OCTA.

In our multivariable logistic regression analysis, systolic blood pressure showed statistically significant predictive value, which is highly consistent with extensive epidemiological studies regarding the relationship between hypertension and DN. In contrast, both diastolic blood pressure and EF/BSA failed to achieve statistical significance, with EF/BSA showing a protective trend but potentially not yet serving as an important predictor of renal function in this early-stage diabetic cohort, this may be attributable to the limitations of the EF/BSA index, which cannot substitute for direct hemodynamic parameters such as cardiac output or systemic vascular resistance in evaluating systemic contributions to microvascular dysfunction. Notably, retinal microvascular parameters—specifically perfusion densities of the superficial and deep plexuses—showed independent associations with renal function, suggesting shared pathophysiological pathways between retinal and renal microvasculature beyond systemic hemodynamics, such as endothelial dysfunction, inflammation, or advanced glycation [3, 4]. Previous studies and meta-analyses corroborate these findings, highlighting the unique value of retinal microvascular assessment for early detection of DN [13–16]. Future prospective studies incorporating direct hemodynamic measurements will be essential to clarify the interaction between systemic and local determinants of microvascular disease.

Our exploratory interaction analysis assessed whether the relationships between OCTA parameters and renal function varied across key clinical subgroups. Although none of the six interaction terms reached statistical significance after multiple comparison correction, this finding provides important clinical insight. The absence of significant interactions indicates that retinal microvascular parameters and renal function show consistent associations across different ages and glycemic control levels. This consistency supports the potential broad applicability of OCTA biomarkers in early-stage diabetic patients, strengthening their clinical utility by suggesting that clinicians would not need to adopt subgroup-specific interpretive thresholds.

As two major microvascular complications of diabetes, DR and DN significantly impact patient quality of life. These conditions are characterized by insidious progression and intricate pathophysiology involving overlapping molecular pathways, such as hyperglycaemia-induced microvascular dysfunction, RAAS hyperactivation, oxidative stress and inflammatory cascades and haemorheological derangements. Our study revealed that, in individuals with diabetes, the eGFR is positively correlated with the retinal microvascular characteristics and choroidal structure in all almost grids and layers, and BUN and the UACR are negatively correlated with the retinal microvascular characteristics and choroidal thickness, especially in the central ring. These results are consistent with the pathophysiological mechanism of DN. As the principal organ of excretion, the kidney maintains homeostasis through blood filtration, urine production, and elimination of metabolic waste. Its glomerular filtration capacity is quantitatively represented by the eGFR, a key metric for assessing renal function. A decrease in the eGFR signifies compromised filtration capacity, leads to urea retention and allows albumin leakage into the urine, resulting in elevated BUN and UACR levels in the serum. Moreover, with that of the DCP, the PD of the SCP was more closely related to renal function indices. This finding is consistent with the findings of Yao et al., who also included patients with diabetes but without DR in their study. This may be attributed to the following points: (1) Superficial vessels are more sensitive to systemic metabolic disturbances due to their role in supplying highly metabolic retinal layers and direct exposure to the vitreous humor. (2) Histologically, the SCP forms an anastomosing network that vascularizes and interconnects adjacent capillary beds, whereas the DCP features distinct lobular configurations with limited interplexal anastomoses [38]. (3) Neurovascular unit interactions play a key role. Superficial vessels have a closer coupling with retinal ganglion cells, and their signaling pathways are more affected by the renin - angiotensin system in diabetes and renal impairment, unlike deep vessels which are more influenced by local factors.

While statistical significance was reported in our research, the clinical relevance remains unclear. A cross-sectional comparison between our correlation coefficient and that of previous studies indicates that our correlation coefficient is similar to those of previous studies [15, 30]. The reasons for the relatively low correlation coefficient are as follows: (1) the progression and prognosis of diabetic nephropathy is influenced by multiple factors, limiting the correlation coefficient of a single factor; (2) sample heterogeneity is prevalent in clinical settings. Despite moderate correlations, the factor’s role in specific subgroups warrants attention.

Recent meta-analyses reveal that traditional markers like microalbuminuria exhibit an AUC of 0.70–0.75 for predicting CKD progression in diabetic patients. Compared with traditional biomarkers such as UACR, OCTA parameters like SCP-PD show only moderate AUC values. Authoritative guidelines including the KDIGO 2020 and Chinese Guidelines for the Primary Management of Diabetic Kidney Disease, have been stipulate that urinary albumin-to-creatinine ratio (UACR) ≥ 30 mg/g and estimated glomerular filtration rate (eGFR) < 60 mL/min/1.73 m² are the core diagnostic criteria. Therefore, OCTA parameters should not be used alone as diagnostic evidence for renal impairment, nor can they replace established markers such as UACR; rather, they should be interpreted in combination with these gold standards. Nevertheless, owing to the unique advantage of being noninvasive, OCTA may have potential value in ‘collaborative diagnostic’ settings. For example, SCP-PD data can be obtained simultaneously during routine retinal OCTA examinations in patients with diabetes—without additional sampling, invasive procedures, or costs—thus achieving high patient acceptance and reducing the risk of missed detection due to limitations of traditional biomarkers such as UACR or as a means of risk re-stratification among patients with mildly elevated UACR. In this way, OCTA is positioned not as a replacement, but as a meaningful adjunct to traditional biomarkers. Therefore, we believe that although the AUC is slightly lower, OCTA, as a non-invasive, convenient and real-time result-generating detection method, still has certain advantages in clinical risk assessment. In addition, we would like to remind readers to pay attention to device differences when interpreting the results. The cut-off values for SCP-PD(C) and DCP-PD(C) were proposed based on the Zeiss PLEX Elite 9000, and these values may vary across different devices [39].

This study enrolled Chinese patients, mainly permanent residents of Shanghai. As a global metropolis, Shanghai’s residents are subject to unique genetic, environmental, and lifestyle determinants. Genetic polymorphisms, such as those in VEGF and aldose reductase genes, modulate vascular integrity and cellular damage, increasing the risk of concurrent ocular and renal complications. Environmental pollutants and urban stressors trigger inflammation and oxidative stress, while dietary habits and sedentary lifestyles further exacerbate disease progression. These factors collectively complicate the interplay between diabetic retinopathy and nephropathy, warranting further large-scale investigations. In studies involving non-Chinese populations, Meng HY et al. reported a significant reduction in VLD and PD in patients with diabetes and CKD [40]. Research teams led by Maria Vadalà [41], Caterina Carollo [42], Leopold Schmetterer [43], and others have also reported correlations between OCTA parameters and renal function. These findings suggest that in non-Chinese populations, OCTA metrics can contribute to monitoring renal function.

Our study has several limitations: (1) while our findings demonstrate robust associations between retinal microvascular parameters and renal function, we acknowledge that the cross-sectional design limits causal inference, the causal mechanism still needs to be further verified through prospective cohort studies or interventional trials. (2) This study was a single-center, single-ethnicity cross-sectional investigation conducted in Shanghai, China, where genetic background, living environment, and healthcare practices are relatively homogeneous. Therefore, whether the observed associations can be generalized to other ethnic groups or healthcare systems remains to be further validated. (3) this study did not include a normal control population, and it is impossible to completely rule out the impact of systemic aging or diabetes-unrelated vascular diseases on the results. In the future, multicenter cohort studies including healthy controls are needed to further verify the specificity of cross-organ damage of retinal OCTA indicators in diabetic and non-diabetic populations (4) The OCTA automated segmentation used in this study was validated against manual expert segmentation. The lack of validation against a histological gold standard remains a major challenge in the field, primarily due to the inherent difficulty in obtaining in vivo histological samples. (5) 7-point SMBG based CV cannot match continuous glucose monitoring (CGM) which provides more comprehensive and continuous data, future research should incorporate CGM for more precise assessment. (6) The clinical “no DR” definition was adopted due to the current absence of validated OCTA-based criteria for preclinical DR. Future work needs establish population-specific OCTA thresholds by correlating detailed parameters with progression data, thus enabling more precise cohort definition. (7) The cross-sectional, correlational design of our study precluded both the proposal of a screening workflow incorporating OCTA parameters with gold standards for renal function prediction and a formal cost-effectiveness analysis. Future multicenter studies with long-term follow-up are planned to develop such workflows and conduct economic evaluations to facilitate clinical translation. (8) These findings must be recognized as device-specific to the PLEX Elite 9000©. Inherent variations in hardware and software between OCTA systems preclude direct comparison of quantitative results.

## Conclusions

Our findings indicate that both the retinal and choroidal structures, along with the retinal microvascular characteristics, are independently associated with renal function in early-stage Chinese DM patients. The retinal microvascular characteristics in the central area measured by SS-OCTA and the choroidal structure are most closely related to renal function and are the most suitable candidates for use as biomarkers. Among the renal function indicators included in our study, the eGFR is the most likely to be predicted by OCTA. OCTA parameters (e.g., SCP-PD) in patients with diabetes but without DR may have supplementary potential for screening early renal-related microvascular abnormalities. The current evidence requires further validation through multicenter cohort studies to confirm cross-device consistency, and long-term follow-up to clarify its predictive value for renal function decline, before its clinical utility as a screening tool can be established.

## Supplementary Information

Below is the link to the electronic supplementary material.

Supplementary Material 1

Supplementary Material 2

## Data Availability

For researchers interested in accessing the anonymized dataset for scientific purposes, we have established a formal request process through our corresponding author. Qualified researchers may contact us directly to request access to the data under appropriate data sharing agreements that ensure patient confidentiality is maintained.
