# Supplementary material for: Correlation between renal function and OCTA parameters of the retina and choroid in early-stage diabetic patients
Source: J Transl Med. 2025 Nov 25;23:1368. doi: 10.1186/s12967-025-07489-w (PMC12670816; doi:10.1186/s12967-025-07489-w)
Supplement: Supplementary file 1 — Supplementary Material 1 [file 12967_2025_7489_MOESM1_ESM.docx]

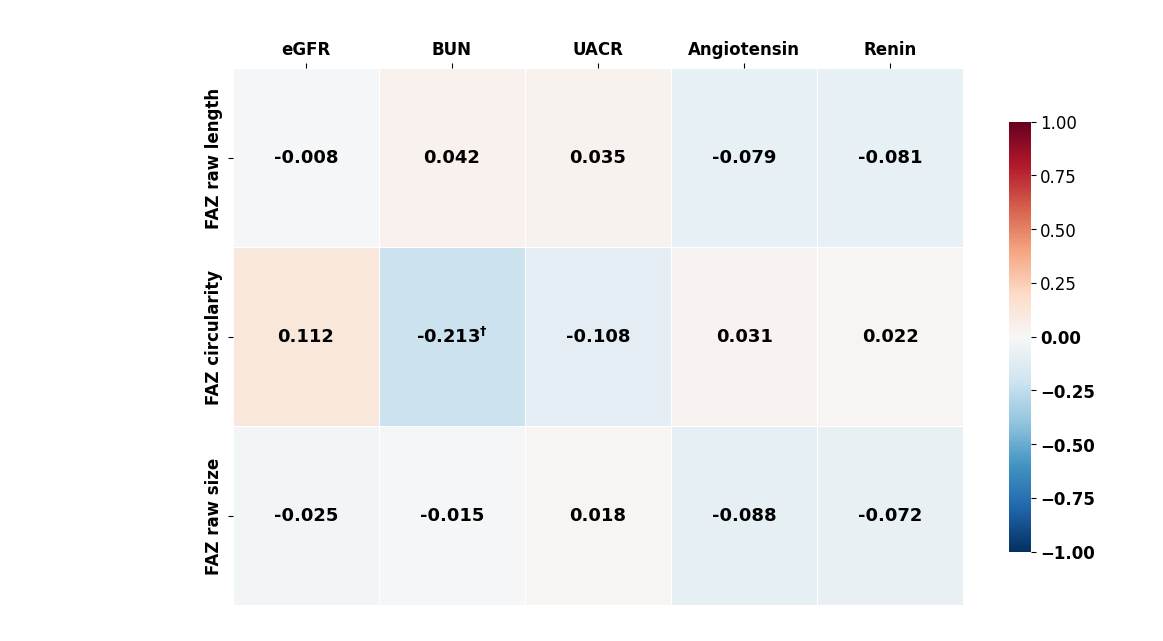


**Figure S1: Correlation between FAZ parameters and renal function indicators**

Figure S1: Heatmap of correlation coefficients between FAZ parameters and renal function indicators. Red indicates positive correlation, blue indicates negative correlation, and the color intensity represents the strength of correlation. To control for multiple comparisons, Benjamini-Hochberg procedure was applied to adjust p values. † indicates correlations that remained significant after FDR correction (q < 0.05)

Abbreviation: FAZ: foveal avascular zone; eGFR: estimated glomerular filtration rate; BUN: blood urea nitrogen; UACR: urinary microalbumin to creatinine ratio.
