# Supplementary material for: Correlation between renal function and OCTA parameters of the retina and choroid in early-stage diabetic patients
Source: J Transl Med. 2025 Nov 25;23:1368. doi: 10.1186/s12967-025-07489-w (PMC12670816; doi:10.1186/s12967-025-07489-w)
Supplement: Supplementary file 2 — Supplementary Material 2 [file 12967_2025_7489_MOESM2_ESM.docx]

Methods：

1. ETDRS Retina Thickness v0.4

Macular thickness (MT) was obtained using the ETDRS Retina Thickness v0.4 algorithm, which measures the distance between the internal limiting membrane (ILM) and the central line of the retinal pigment epithelium (RPE). First, the algorithm automatically segments the ILM and RPE layers within an OCT-A volume as surfaces, leveraging common tools in PLEX Elite review software. The distance between these surfaces (in microns) generates a retina thickness topographic map across the scan's field of view (FOV).​

In the second step, the algorithm locates the fovea center, the Optic Nerve Head (ONH) center, and the ONH - corresponding region, again using tools from PLEX Elite review software. Once correctly identified, the ONH region is excluded from the retina thickness analysis. Based on the fovea center location and scan laterality (left or right eye), the ETDRS grid is positioned within the scan's topographic span. Finally, average thickness values for each ETDRS grid region are calculated.

The schematic diagram is as follows：


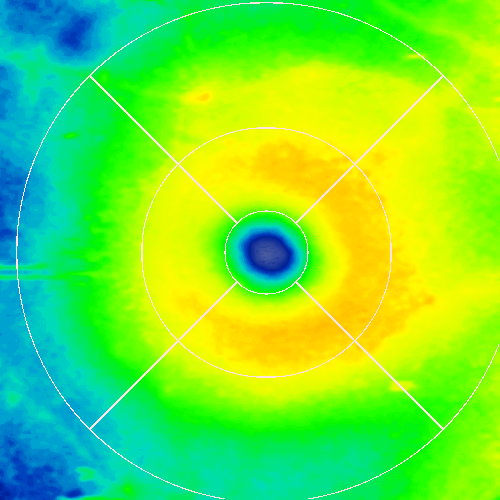


1. Macular Density v20210824-B

The perfusion density of the SCP (SCP-PD) and DCP (DCP-PD); the vessel length density of the SCP (SCP-VLD) and DCP (DCP-VLD); and the raw length, circularity, and raw size values of the FAZ were obtained from the Macular Density v20210824-B algorithm.

Image Re - creation and Quantification​

The algorithm generates slab images of the retina, including the superficial and deeper layers, without projection artifacts. The angio slabs of the retina (ranging from the top of the inner limiting membrane (ILM) to 41 microns below Bruch's membrane), the superficial layer (from the top of the ILM to the inner plexiform layer (IPL)), and the deep layer (from the top of the IPL to the outer plexiform layer (OPL)) serve as the foundation for subsequent quantification.

Perfusion Density​

Perfusion density is defined as the ratio of the total area of perfused vasculature to the unit area within a measurement region. It is expressed as a value ranging from 0 (indicating no perfusion) to 1 (representing full perfusion). Due to the inherent presence of non-perfused regions between vessels, perfusion density values generally stay below 0.5.​

Vessel Length Density​

Vessel length density is defined as the total length of perfused vasculature per unit area in a given measurement region, with the unit of measurement being inverse millimeters. Conceptually, it is equivalent to measuring the total length of all untangled retinal vasculature with a tape measure and then dividing this length by the original occupied area. The resulting value has a minimum of 0 (indicating the absence of vessels) and an unbounded maximum. Typically, vessel length density values remain below 30 inverse millimeters.​

The schematic diagram is as follows：


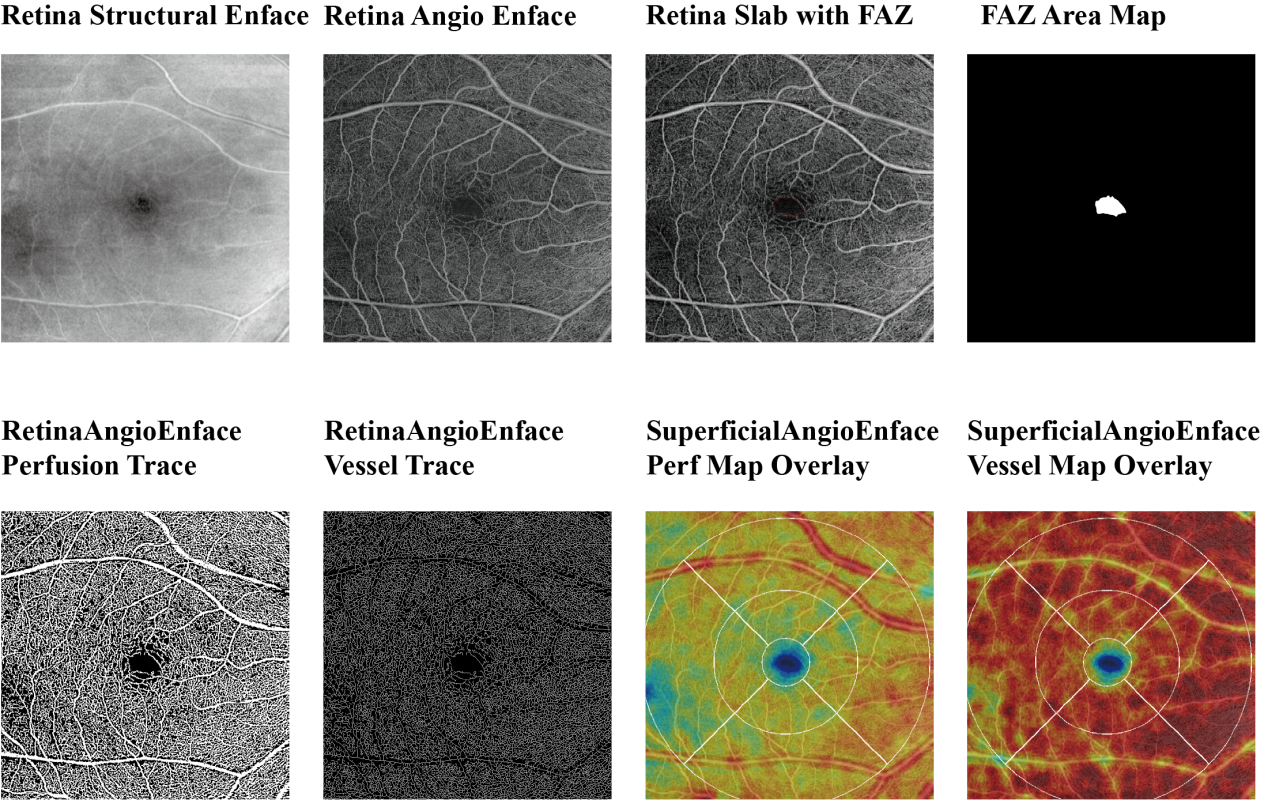


1. Choroid quantification v20220224-B

Choroidal volume (CV), choroidal thickness (CT), and choroidal vasculature index (CVI) data were obtained using the Choroid Quantification v20220224-B algorithm. This algorithm measures CT and CV within extended ETDRS sectors, defining the choroid as the region between Bruch's membrane and the choroidal-scleral interface, with boundaries determined via Multilayer Segmentation. Any manual segmentation edits made on the instrument or review software prior to data upload are incorporated into the analysis, and the optic nerve head (ONH) location is automatically excluded from calculations. CVI, computed from structural cube data, represents the proportion of local choroidal volume occupied by vessel volume.​

To validate CVI accuracy, an experienced ophthalmologist (SL) manually marked choroidal luminae in multiple B-scan images using ImageJ. The results showed a high degree of concordance between the built-in algorithmic calculations and manual measurements.

The schematic diagram is as follows：


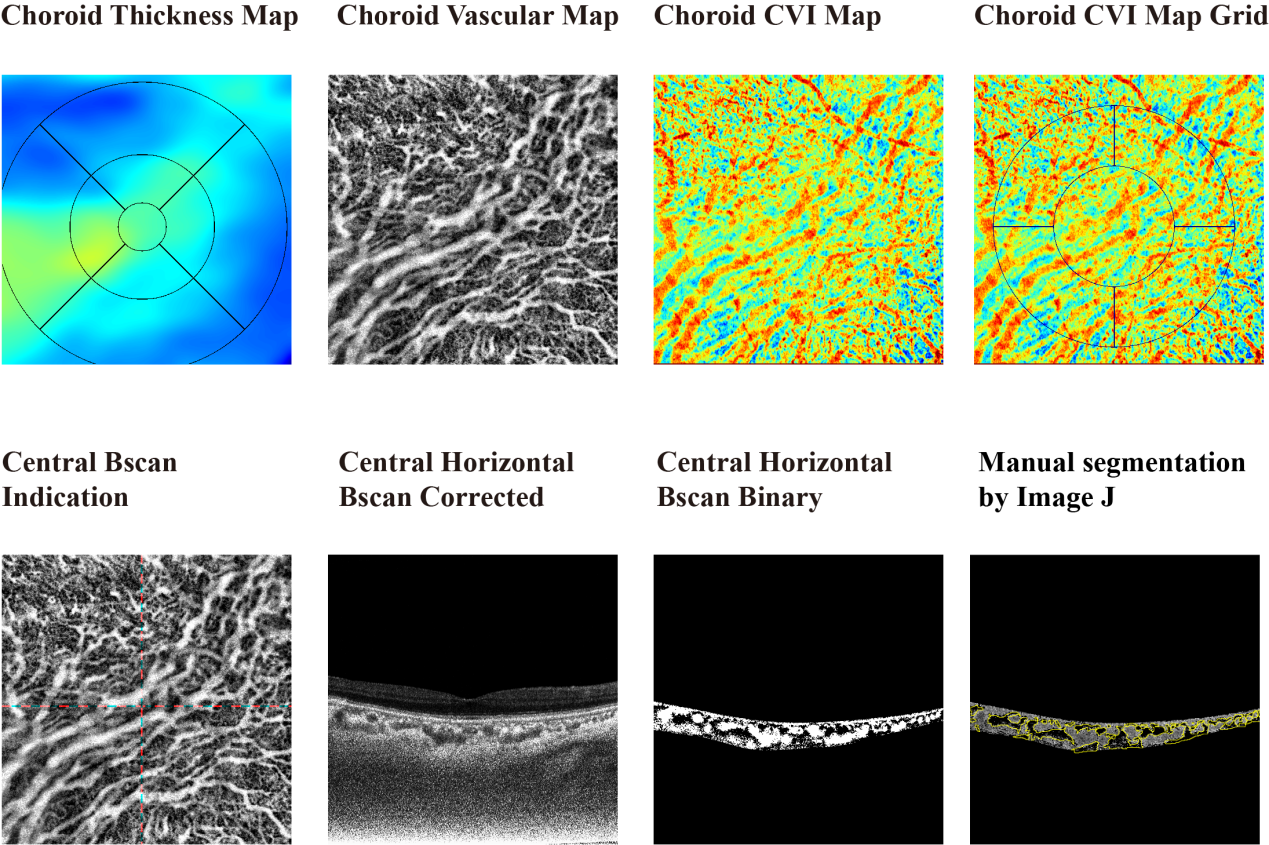


1. We calculated Dice similarity coefficients, intraclass correlation coefficients, and performed Bland-Altman analysis to assess agreement and systematic bias.

Table S1 Validation Results for Automated Choroidal Segmentation (N=50)

| **Comparison Groups** | **Dice Similarity Coefficient** | **Mean CVI Difference** | **ICC** | **p-value** |
| --- | --- | --- | --- | --- |
| **Automated vs. Grader 1 (SL)** | **0.827 ± 0.033** | **-0.013 ± 0.017** | **0.917** | **<0.001** |
| **Automated vs. Grader 2 (XXD)** | **0.874 ± 0.045** | **-0.019 ± 0.021** | **0.892** | **<0.001** |
| **Grader 1 vs. Grader 2** | **0.929± 0.068** | **0.012± 0.016** | **0.931** | **<0.001** |

1. Table S2 VIF for logistic regression analysis.

| **Variable Indicators** | **Category/Unit** | **VIF** | **Variable Indicators** | **Category/Unit** | **VIF** |
| --- | --- | --- | --- | --- | --- |
| **Age** | **<50 years** | **-** | **AL** | **per mm** | **1.345** |
|  | **≥50 years** | **2.483** | **SE (Spherical Equivalent)** | **Emmetropia (-0.50D ≤ SE ≤ +0.50D)** | **-** |
| **Sex** | **Female** | **-** |  | **Mild myopia (-3.0D < SE < -0.50D)** | **1.267** |
|  | **Male** | **1.156** |  | **Moderate myopia (-6.0D <SE ≤ -3.0D)** | **1.361** |
| **BMI** | **<24 kg/m²** | **-** |  | **Hyperopia (SE > +0.50D)** | **1.867** |
|  | **≥24 kg/m²** | **1.120** | **CRP** | **per mg/L** | **2.876** |
| **HbA1c** | **<7%** | **-** | **ESR** | **per mm/h** | **2.652** |
|  | **≥7%** | **1.082** | **IL-6** | **per pg/mL** | **2.451** |
| **Glycemic Variability** | **per 1 SD** | **1.423** | **PCT** | **per ng/mL** | **4.347** |
| **Diabetes Duration** | **<10 years** | **-** | **SCP-PD (C)** | **per 1%** | **1.292** |
|  | **≥10 years** | **4.328** | **DCP-PD (C)** | **per 1%** | **1.288** |
| **Dyslipidemia** | **No** | **-** | **CV (Overall)** | **per mm³** | **3.371** |
|  | **Yes** | **3.245** | **RAAS Inhibitor Use** | **No medication use** | **-** |
| **SBP** | **per 10 mmHg** | **4.045** |  | **RAAS inhibitor** | **1.285** |
| **DBP** | **per 10 mmHg** | **3.023** |  | **Hypolipidemic agents** | **2.239** |
| **EF/BSA** | **per unit** | **1.189** |  | **Bose use** | **1.193** |
